# Surgical and Non-Surgical Procedures Associated with Recurrence of Periodontitis in Periodontal Maintenance Therapy: 5-Year Prospective Study

**DOI:** 10.1371/journal.pone.0140847

**Published:** 2015-10-23

**Authors:** Fernando Oliveira Costa, Luís Otávio Miranda Cota, José Roberto Cortelli, Sheila Cavalca Cortelli, Renata Magalhães Cyrino, Eugênio José Pereira Lages, Ana Paula Lima Oliveira

**Affiliations:** 1 Department of Periodontology, Dentistry School, Federal University of Minas Gerais, Belo Horizonte, Minas Gerais, Brazil; 2 Center for Periodontal Research, University of Taubaté, Taubaté, São Paulo, Brazil; 3 Department of Periodontology, Dentistry School, Federal University of Uberlândia, Uberlândia, Minas Gerais, Brazil; University of North Carolina at Chapel Hill, UNITED STATES

## Abstract

**Background and Objective:**

Prospective studies that investigated the influence of surgical and nonsurgical procedures in the recurrence of periodontitis and tooth loss in periodontal maintenance therapy (PMT) programs have not been previously reported. The objective of this study was to evaluate longitudinally the recurrence of periodontitis in regular compliers (RC) and irregular compliers (IC) individuals undergoing surgical and non-surgical procedures over 5 years in a program of PMT.

**Materials and Methods:**

A total of 212 individuals participated in this study. Full-mouth periodontal examination including bleeding on probing, probing depth, and clinical attachment level were determined at all PMT visits over 5 years. The recurrence of periodontitis was evaluated in RC and IC individuals undergoing surgical and non-surgical procedures in PMT. The influences of risk variables of interest were tested through univariate analysis and multivariate logistic regression.

**Results:**

Recurrence of periodontitis was significantly lower among RC when compared to IC. Individuals with recurrence of periodontitis and undergoing surgical procedures showed higher probing depth and clinical attachment loss than those who received non-surgical procedures. Recurrence of periodontitis was higher in individual undergoing surgical procedures and irregular compliance during PMT.

**Conclusions:**

Irregular compliance and surgical procedures in individuals undergoing PMT presented higher rates of recurrence of periodontitis when compared to regular compliant patients undergoing non-surgical procedures.

## Introduction

The benefits of periodontal maintenance therapy (PMT) in maintaining the homeostasis of periodontal tissues obtained after active periodontal therapy (APT), which includes surgical and non-surgical procedures, has been extensively documented in numerous studies [1–6].

A classic problem in PMT programs is difficulty in maintaining the patient’s compliance and in scheduling regular maintenance visits [1–4]. The frequency of visits in the literature conflicts greatly, with the intervals between visits considered as regularly as 3–4 months [1,5–7], 6 months [8,9],or a maximum of 12 months when considering the risk profile of the individual [1,2,10–12].

However, without establishing a regular program of clinical re-evaluation, adequate biofilm control, and reinforcement of oral hygiene instructions, the benefits of PMT cannot be maintained [3–7,13]. Several indicators or risk factors and biological, behavioral, and social conditions can influence the status of individuals undergoing PMT [1,3,12–14].

PMT visits can be considered a critical factor for success in controlling periodontal disease. Moreover, neglecting a regular PMT program has been associated with a higher risk of recurrence and with the progression of periodontitis [1,3–5].

The efficiency of surgical and non-surgical periodontal procedures in the context of periodontal therapy has been widely reported [15–19] but scarcely in PMT. Furthermore, recurrence of periodontitis and tooth loss (TL) has been reported in studies in the absence of periodontal treatment [18,20,21], as well as in many PMT retrospective studies [7,8,22–26] and in relatively few prospective studies [3,5,27,28].

In addition, few prospective studies to date on PMT have reported a direct influence of the performance of surgical and non-surgical procedures on the recurrence of periodontitis associated with the degree of compliance of individuals.

Thus, the objective of this study was to evaluate longitudinally the recurrence of periodontitis in regular and irregular compliance individuals undergoing surgical and non-surgical procedures over 5 years in a program of PMT. In addition, the influence of biological and behavioural risk variables on this association was investigated.

## Material and Methods

The sample of this prospective cohort study comprised 265 individuals, age 23–70 years old, who were included in a PMT program, and monitored in a private dental clinic in Belo Horizonte city, Brazil (from August 2006 to February 2014) over a 5-year period in consecutive recalls for PMT visits.

Participants were informed of the aims of the study and provided written informed consent prior to their participation in the study. The present study and all protocols were approved by the Research Ethics Committee of the Federal University of Minas Gerais, Brazil (protocol #060/05). Subjects’ rights were protected at all times.

Initially, subjects in this cohort with good general health, except for the presence of diabetes type 2, who had undergone APT (comprised of non-surgical and/or surgical procedures) were included in the sample. In addition, included subjects also presented the following criteria: (a) diagnosis of chronic moderate to advanced periodontitis based on Armitage [29]; (6) prior to APT, with at least 4 sites with probing depth (PD) ≥5 mm and clinical attachment loss (CAL) ≥3 mm, bleeding on probing (BOP), and/or suppuration (SU), and radiographic evidence of bone loss; (b) completion of APT in a period of less than 4 months prior to entry into the PMT program; and (c) at least 14 teeth in the oral cavity (3–5,28).

Individuals were excluded from the study if they: (a) were pregnant (n = 3); (b) had debilitating diseases that could impair the immune system (such as HIV/AIDS, cancer, and auto-immune diseases; n = 3); (c) presented with drug-induced gingival hyperplasia (n = 4); (d) presented with type 1 diabetes (n = 3); (f) had less than 14 teeth present (n = 10); or (g) refused to participate (n = 30).

Based on the above criteria, a convenience sample of 212 volunteers was eligible for this study. According to the pattern of compliance during PMT visits, subjects from this cohort were determined to be regular compliers (RC; N = 96 subjects were 100% compliant for PMT visits, with a maximum interval of 6 months) and irregular compliers (IC; N = 116 subjects that missed any of the PMT visits but continued to appear on an irregular basis, with a maximum interval of 18 months).In this study, the baseline was considered after APT (first visit to PMT) and the final exam last recorded as having been performed under PMT.

Data regarding gender, age, co-habitation status, educational level, smoking (smokers/former smokers—reporting having smoked more than 100 cigarettes throughout their lives—and non-smokers) [30, 31], and diabetes (fasting plasma glucose levels >126 mg/dl or taking anti-glucose for more than 2 weeks–ADA) [32] were collected for all of the subjects. All collected data that might have presented temporal changes were confirmed at the last PMT visit.

During maintenance visits, a complete periodontal examination was performed, and data regarding plaque index (PI) [33], PD, CAL, BOP, furcation involvement, and SU were recorded. All periodontal parameters were used to determine periodontal status in 4 sites per tooth. Examinations were performed with a manual periodontal probe (PCPUNC15BR and PQ2NBR, Hu-Friedy®, Chicago,USA). Methodology for data collection and periodontal clinical procedures during all PMT visits were the same as reported by Lorentz et al. [3].

### Clinical examinations

During the PMT visits, the following procedures were performed: (a) interviews, to determine possible changes in variables of interest (demographic, biologic, and behavioral); (b) periodontal evaluation, through clinical parameters previously described; (c) application of disclosing agents and oral hygiene instructions, using the Bass technique, as well as interproximal toothbrushes and dental floss; (d) coronal prophylaxis and fluoride application; and (e) mechanical non-surgical or surgical debridement, when appropriate. Surgical treatment was performed in the absence of positive response (i.e, PD ≥ 5 mm with BOP) after procedures of sub-gingival scaling and root planning under anesthesia. Surgery treatment needs were evaluated after 45 to 60 days of non-surgical therapy. Regenerative techniques and use of biomaterials were not used in surgical procedures.

### Determination of recurrence of periodontitis and retreatment needs

Sites with recurrence of periodontitis after APT, that is showing PD ≥4mm and CAL≥3 mm, together with the persistence and/or presence of BOP and/or SU, during any of the subsequent recall evaluations [3,12],were determined as having retreatment needs. Individuals diagnosed with recurrence or having sites with residual probing depth changes were first re-treated with mechanical subgingival debridement (non-surgical treatment). After periodontal re-evaluation (45 to 60 days), sites with persistent PD ≥4mm and CAL≥3 mm underwent surgical procedures (preferably Widman modified flap surgery).

### Inter- and intra-examiner calibration

All of the interviews, examinations, and clinical periodontal procedures were performed by 2 trained and calibrated periodontists (F.O.C. and E.J.P.L). Measurements of PD and CAL were recorded and repeated within a 1-week interval for 10 subjects randomly selected from both groups at baseline and at the final examination. The data were tested with a non-parametric kappa test and intra-class correlation. The kappa coefficients for both intra- and inter-examiner, as well as intra-class correlation coefficients, were greater than 0.87.

Prior to the beginning of the study, a training process was conducted to standardize the application of smoking questionnaires during the interviews. Interviews were repeated with 12 subjects to verify the quality of the categorical data obtained. In addition, all of the data collected by the questionnaire that might have presented temporal changes were confirmed at each PMT visit.

### Statistical analysis

The statistical analysis included a descriptive characterization of the sample according to variables of interest. Group comparisons by means of the chi-squared test, ANOVA, and Student’s *t*-test were performed when appropriate. When equal variances were assumed, the variables were compared by means of ANOVA and Bonferroni’s post-hoc test. When equal variances were not assumed, variables were compared by means of Welch and Tamhane post-hoc test.

The changes in clinical parameters over time were tabulated and summarized according to treatment group (surgical and non-surgical procedures) (S1 Dataset). The analysis was performed for the total sample, including individuals, teeth, and sites. Subsequently, the analysis was dichotomized for molar and non-molar teeth. The analysis included teeth and sites present at baseline and at the final examination.

Recurrence of periodontitis was assumed on individuals as the unit of analysis based on the proposed definitions. A logistic regression analysis was performed to investigate the association between the recurrence of periodontitis for the following independent predictor risk variables: gender (male/female), age (up to 40; 41/55 and >55 years old), co-habitation status (companion/ no companion), smoking (smokers/former smokers, and non-smokers), number of PMT visits, diabetes, compliance (regular or irregular), plaque index, surgical treatment (need for at least 1 periodontal surgery during the 5 years at any visit to the PMT), BOP (in more than 30% of sites), PD between 4 and 6 mm in up to 10% of sites, and CAL ≥ 3 mm in 30% of sites. The cut off points periodontal variables used in the logistic regression analysis were determined by means of sensitivity and specificity tests. Those within the most appropriate area under the operating characteristic curve (AROC) were adopted (higher values of specificity and sensitivity). Thus, the following cut-off points were established: BOP in more in up to 30% sites (AROC = 0.85); PD 4 -6mm in up to 10% sites (AROC = 0.87); CAL in up 30% (AROC = 0.82.)

All of the variables included in the final multivariate model were determined to be independent through the assessment of their co-linearity. Odds ratio estimates and their confidence intervals were calculated and reported.

In a posteriori power analysis calculation, irregular compliance was determined to be the exposure and recurrence of periodontitis was determined to be the outcome. Therefore, the incidence in exposed individuals was of 0.36, the incidence in unexposed individuals was of 0.12, and the relative risk was of 3.0. Sample in the present study (n = 212) provided a power of 0.987. All of the tests were performed using statistical software SPSS 16.0 (Statistical Package for Social Sciences, Chicago, USA). The results were considered significant if a *P* value less than 5% was attained (*P*<0.05).

## Results

Characteristics of the sample regarding social, demographic, and biological variables of interest were evaluated (S1 Table). Sample characteristics were also described in a previous study [4]. Significant differences were reported only in PMT visits, age (41 to 55 years), and gender in relation to the RC and IC groups, showing a homogeneous sample.

The periodontal status of the sample at baseline (first PMT visit) and at the final examinations is presented in Table 1. In general, no significant differences were observed with regard to the parameters plaque index, PD, and CAL between the groups at the baseline examination. As a result, both groups were determined to be homogeneous following APT. Nevertheless, significant differences between the RC and IC groups were observed at the final examination. The IC group exhibited a significantly higher PI, mean of BOP, PD, CAL, and TL. During the 5 years of PMT, the IC and RC groups lost, respectively, a mean of 0.6 teeth (0.12 teeth lost/year) and 1.8 (0.3 6 teeth lost/year).

**Table 1:** Periodontal status of regular compliers (RC) and irregular compliers (IC) groups at baseline and final examination.

	Baseline (after active periodontal therapy)				Final Examination			
	RC (n = 96)		IC(n = 116)		RC(n = 96)		IC(n = 116)	
Periodontal Parameter	Mean	(±)s.d	Mean	(±)s.d	Mean	(±)s.d	Mean	(±)s.d
**Probing depth (sites** * **)**								
**≥ 4-5mm**	1.5Aa	± 3.7	1.7Aa	± 3.5	3.5Ab	± 4.1	4.1Bb	± 3.8
**≥ 6mm**	0.5Aa	± 1.8	0.7Aa	± 1.7	0.9Ab	± 0. 3	1.5Bb	± 0.5
**Clinical attachment loss (sites** * **)**								
**≥ 4-5mm**	13.2Aa	± 1.5	13.4Aa	± 1.5	12.9Ab	± 1.5	14.1Bb	± 2.1
**≥ 6mm**	9.9Aa	± 0.9	10.2Aa	± 1.5	8.1Aa	± 1.3	13.8Bb	± 1.5
**Bleeding on probing (sites** * **)**	24.6Aa	± 4.2	27.8Ba	± 6.1	24.9Ab	± 5.1	32.8Bb	± 6.9
**Suppuration (sites** * **)**	0.09Aa	± 0.12	0.11Aa	± 0.18	0.04Ab	± 0.01	0.17Bb	± 0.06
**Plaque Index**	33.4Aa	± 4.7	34.1Aa	± 5.9	35.25Ab	± 5.23	44.98Bb	±7.34
**Mean number of teeth lost**	3.8Aa	-	4.0Aa	-	4.4Ab	-	5.8Ab	-

*Mean % of affected sites ± standard deviation

Comparisons between groups within each examination time (baseline RC versus baseline IC, and final RC versus final IC) followed by distinct capital letters are significant different (Student-t test for independent samples). Comparisons between examination times within each group (baseline RC versus final RC, and baseline IC versus final IC) followed by distinct lower case letters are significant different (Student-t test for independent samples). Multiple comparisons adjusted by Bonferroni correction (p≤0.02).

In the RC group, 25 (26.0%) individuals, 90 (4.0%) teeth, and 243 (2.7%) sites presented recurrence of periodontitis, while in the IC group 42 (36.2%) individuals, 165 (6.07%) teeth, and 468 (4.888%) sites, presented recurrence of periodontitis.

In cases of recurrence of periodontitis, surgical treatment was performed, when necessary, in the RC and IC groups, in 12 and 25 individuals, 38 and 96 teeth, and 138 and 294 sites, respectively, while non-surgical procedures were performed in the RC and IC groups on 13 and 17 individuals, 52 and 69 teeth, and 105 and 174 sites, respectively. Surgical treatment was performed significantly more frequently in molar teeth in both groups, with IC > RC. Non-surgical treatment was more frequent in non-molars, with RC > IC. Individuals undergoing surgical procedures have more TLthan those who underwent non-surgical procedures, with IC > RC. It is important to note that most sites in both groups responded favorably to conservative mechanical (supra-gingival) debridement procedures (Table 2).

**Table 2:** Recurrence of periodontitis and non-surgical or surgical procedures performed during PMT (full-mouth, non-molar and molar) in RC and IC groups.

	During PMT (RC *versus* IC)	
Global sample	RC (Individuals = 96; Teeth = 2,217; Sites = 8,868) n (%)	IC (Individuals = 116; Teeth = 2,436; Sites = 9,744) n (%)
**Recurrence of periodontitis**		
Individuals	25 (26.0)a	42 (36.2)b
Teeth	90 (4.0)a	165 (6.7)b
Sites	243 (2.7)a	468 (4.8)b
**Surgical Treatment in Recurrence of Periodontitis in full-mouth**		
Individuals	12 (48.0)a	25 (59.5)b
Teeth	38 (42.2)a	96 (58.2)b
Sites	138 (56.7)a	294 (62.8)b
**Non-surgical Treatment in Recurrence of Periodontitis in full-mouth**		
Individuals	13 (52.0)a	17 (40.5)b
Teeth	52 (57.8)a	53 (32.1)b
Sites	105 (43.3)a	174 (37.2)b
**Surgical Treatment in Recurrence of Periodontitis in non-molar sites**		
Individuals	4 (16.0)a	9 (21.4)b
Teeth	8 (8.9)a	28 (16.9)b
Sites*	24 (9.8)a	44 (9.4)a
**Non-surgical Treatment in Recurrence of Periodontitis in non-molar sites**		
Individuals	9 (36.0)a	12 (28.6)b
Teeth	38 (42.2)a	41 (24.8)b
Sites	88 (36.2)a	152 (32.4)b
**Surgical Treatment in Recurrence of Periodontitis in molar sites**		
Individuals	8 (32.0)a	16 (38.0)b
Teeth	30 (33.3)a	68 (41.2)b
Sites	114 (46.9)a	250 (53.4)b
**Non-surgical Treatment in Recurrence of Periodontitis in molar sites**		
Individuals	4 (16.0)a	5 (11.9)b
Teeth	14 (15.5)a	12 (7.3)b
Sites	17 (6.9)a	22 (4.7)b

Comparisons between RC and IC followed by different letters are significant(p <0.05).

The periodontal status of individuals with recurrences of periodontitis at the final examination, with surgical or non-surgical treatment during follow-up, is shown in Table 3.

**Table 3:** Periodontal status of individuals with recurrence of periodontitis with surgical or non-surgical procedures during PMT at final examination.

	RC (recurrence = 25)				IC (recurrence = 42)			
	Surgical (n = 12)		Non-Surgical (n = 13)		Surgical (n = 25)		Non-Surgical (n = 17)	
Periodontal Parameters	Mean	(±)s.d	Mean	(±)s.d	Mean	(±)s.d	Mean	(±)s.d
**Probing depth (sites** * **)**								
**≥ 4-5mm**	2.9 Aa	± 2.9	3.2 Aa	± 3.1	4.2 Ab	± 3.5	4.4 Ab	± 3.8
**≥ 6mm**	0.9 Aa	± 1.4	1.1 Aa	± 1.4	1.4 Ab	± 0. 3	1.6 Ab	± 0.4
**Clinical attachment loss (sites** * **)**								
**≥ 4-5mm**	13.7 Aa	± 1.0	12.9 Ba	± 1.7	14.7 Ab	± 1.2	13.9 Bb	± 2.2
**≥ 6mm**	9.3 Aa	± 0.9	9.1 Aa	± 1.1	13.9 Ab	± 1.3	13.1 Bb	± 1.6
**Bleeding on probing (sites** * **)**	24.2 Aa	± 3.1	24.9 Aa	± 3.4	32.6 Ab	± 3.9	31.9 Ab	± 5.2
**Suppuration (sites** * **)**	0.13 Aa	±0.07	0.14 Aa	± 0.10	0.16 Ab	± 0.03	0.31 Bb	± 0.05
**Plaque Index**	35.4 Aa	± 3.7	36.3 Aa	± 4.9	44.25 Aa	± 6.11	40.78 Bb	±7.14
**Mean number of teeth lost**	4.6Aa		4.1Ba		6.8Ab		6.2Bb	

*Mean % of affected sites ± standard deviation

Comparisons between treatment modalities within each group (RC surgical versus RC non-surgical, and IC surgical versus IC non-surgical) followed by distinct capital letters are significantly different (Student-t test for dependent samples). Comparisons between groups within each treatment modality (RC surgical versus IC surgical, and RC non-surgical versus IC non-surgical) followed by distinct lower cases letters are significantly different (Student-t test for independent samples). Multiple comparisons adjusted by Bonferroni correction (p<0.04).

**Fig 1:**
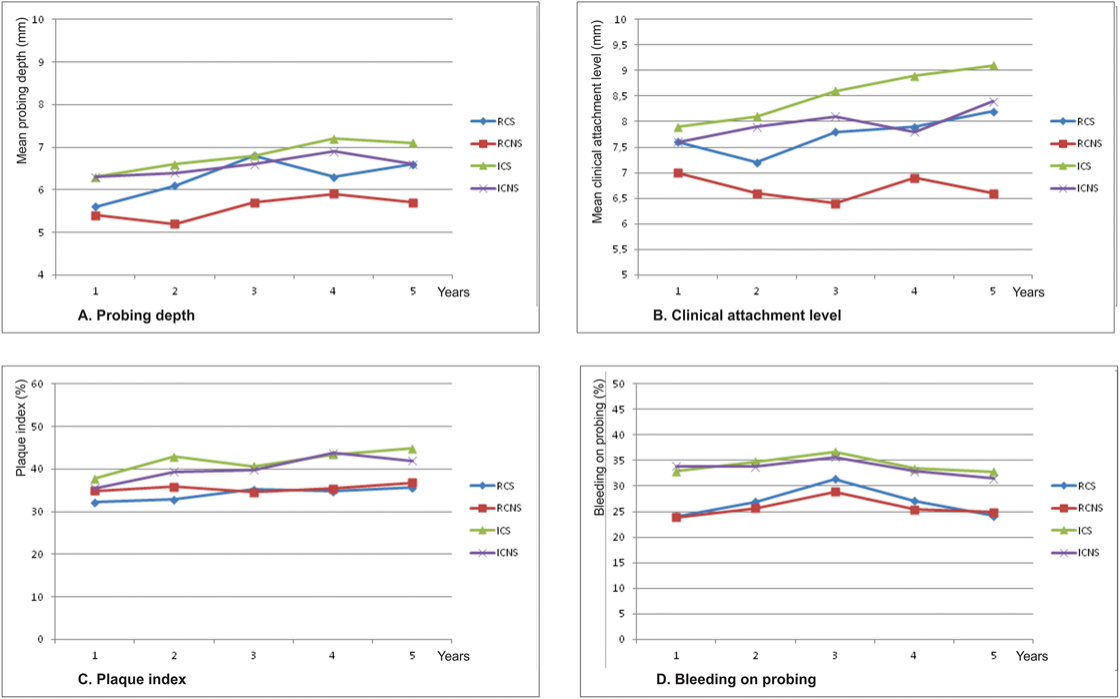
Changes in PD, CAL, PI, and BOP at 1-to-5 years (mean per individuals) by surgical or non-surgical procedures in RC and IC individuals with recurrence of periodontitis. Probing depth and Clinical attachment level (Fig 1a and Fig 1b): IC>RC;IC Surgical (ICS) >IC Non-Surgical (ICNS) = RC Surgical (RCS) >RC Non-Surgical (RCNS)^*^; Plaque index (Fig 1c): IC>RC; ICS >ICNS and RCS = RCNS^**^; Bleeding on probing (Fig 1d): IC>RC; RCS = ICS and ICS = ICNS^*^; ^*^Statistically significant increase in PD, CAL, and BOP per years, using Welch test and Tamhane post hoc analysis (p<0.05). ^**^Statistically significant increase in plaque index per years, using ANOVA and Bonferroni post hoc analysis (p<0.05).

Changes in PD, CAL, BOP, and PI over time (per year) by surgical or non-surgical treatment in RC and IC individuals with periodontitis recurrence are show in Fig 1A, 1B, 1C and 1D. For PD (Fig 1A) and CAL (Fig 1B), it was observed that individuals with recurrence of periodontitis and undergoing surgical procedures showed higher PD and CAL values than those who received non-surgical procedures. These values were higher in the IC group.

With regard to PI (Fig 1C), individuals in the IC group had significantly higher PI values than individuals in the RC group. Individuals in the IC group who were submitted to surgical procedures had significantly higher PI values than those who received non-surgical procedures. Furthermore, individuals in the RC group who were submitted to surgical or non-surgical procedures did not have significant differences in PI values.

In both groups, a greater reduction in BOP (Fig 1D) occurred in the 1st year, and higher values were observed at 3 years (IC = 36.7% and RC = 31.4%). Individuals in the IC group showed higher values for BOP than individuals in the RC group over the 5 years of the study. However, there were no significant differences in the values for BOP in the RC and IC groups, with regard to the type of treatment performed, i.e., surgical or non-surgical.

Final multivariate logistic regression model for recurrence of periodontitis adjusted for all variables of interest is shown in Table 4. The following variables were significantly retained in the model: smoking (OR = 3.8, 1.18–9.67); diabetes (OR = 2.5, 1.05–6.21); BOP in more than 30% of sites (OR = 2.9, 1.53–12.1); PD 4–6 mm in up to 10% of sites (OR = 3.3, 1.13–5.87); irregular compliance (OR = 3.4, 1.24–8.28); and surgical treatment (OR = 2.1, 1.07–6.68).

**Table 4:** Final logistic regression model for the recurrence of periodontitis at final examinations.

Periodontitis Recurrence Logistic Final Model	p	Odds Ratio	95% CI
Smoking	0.002	3.8	1.18–9.67
Diabetes	0.005	2.5	1.05–6.21
BOP more than 30% of sites	0.003	2.9	1.53–12.1
PD 4–6 mm up to 10% of sites	0.011	3.3	1.13–5.87
Irregular compliance	0.002	3.4	1.24–8.28
Surgical treatment	0.033	2.1	1.07–6.68
Constant	<0.001	-	-

BOP = bleeding on probing; PD = probing depth; CI = confidence interval.

## Discussion

The present study showed that most individuals responded favorably to surgical and non-surgical procedures during PMT. However, the IC group exhibited higher PI, mean of BOP, PD, CAL, and TL. Recurrence of periodontitis was significantly higher in the IC group compared to the RC group in all sample units of analysis, i.e., individuals, teeth, and sites. Thus, our results are consistent with previous studies’ findings that in non-cooperating individuals, the disease can recur and lead to TL[3,4,12,26,34,35].

Thus, even with reasonable intervals between PMT visits and surgical and/or non-surgical procedures according to the needs of the individual, recurrence and TL occurred in all groups and can be interpreted as undesirable over the 5 years of monitoring. Corroborating these findings, Renvert & Persson [1] reported on an extensive review that the results obtained after APT cannot be sustained with standard PMT visits between 3–6 months and 3 years, so it is essential to perform individual risk assessment to establish the frequency of visits for PMT.

Dental mortality can be considered an indisputable failure of periodontal treatment [1,4].In our study, the IC and RC groups lost, respectively, a mean of 0.6 teeth(0.12 teeth lost/year)and 1.8 teeth (0.36 teeth lost/year). This finding was similar to previous results from retrospective and prospective studies of PMT, which reported that a 2–3 times higher tooth loss/year occurred in subjects treated for periodontitis who did not comply with maintenance care and up to a 6 times higher loss in individuals not undergoing treatment [3,5,12,20–22,25].Interestingly, during PMT, surgical procedures influenced greater TL after 5 years in RC and IC individuals.

However, several authors [1,28,36–39] have pointed out that most studies comparing surgical versus non-surgical therapy are heterogeneous because they have been conducted comparing different techniques and populations, with too many different designs and follow-up times and no intended treatment; thus, they have reported different results.

In contrast, studies on the influence of surgical or non-surgical modalities after active therapy in PMT programs are scarce and, when available, were performed with retrospective designs [1], small samples, and short periods of observation [1,3,40,41].

Harrel et al. [18]in a retrospective study with the objective of evaluating the effect of no periodontal treatment, non-surgical treatment only, and a combination of non-surgical and surgical treatment on the progression of periodontitis, showed that surgical treatment yielded much more favorable results than non-surgical treatment alone. The authors concluded that compliance with periodontal maintenance recommendations could be a major contributor to this finding. In addition, the authors also recognized that non-surgical procedures were more common in non-cooperative individuals with future recommendations for PMT.

In the present study, it was observed that individuals with recurrence of periodontitis and undergoing surgical procedures showed higher PD and CAL values than those who underwent non-surgical procedures. These results suggest the effectiveness of non-surgical procedures for PMT, especially among RC individuals since higher values for PD and CAL were reported in IC individuals. As already reported, there are few prospective studies in the literature with data on the effectiveness of different treatment modalities for PMT, so the discussion of our results must be limited.

Surgical treatment was performed significantly more frequently in molar teeth in both groups, similar to previous studies [39–42]. Non-surgical treatment was more frequent in non-molars, with RC > IC. The literature consistently reports that surgical periodontal therapy is more often performed in non-molar teeth compared to the molars [42,43]. Clearly these findings reflect a greater difficulty in correct root instrumentation only with non-surgical procedures in molar teeth, compared to non-molars [15,19,39,43]. A major factor for these findings is the morphology of the posterior teeth, especially when associated with furcation lesions [19,42].

In addition, this study showed that the degree of cooperation was positively correlated with a greater need for non-surgical procedures in PMT. This finding is inverse to that described by Harell et al. [18] and similar to a retrospective study [42] evaluating molar teeth of 106 individuals.

In individuals with recurrence of periodontits in the RC group, being submitted to surgical or non-surgical procedures did not result in significant differences in PI. However, worse oral hygiene was observed in IC individuals, especially when undergoing surgical procedures. With regard to the accumulation of plaque after treatment, there is insufficient evidence showing differences between surgical and non-surgical treatments or between various surgical procedures [39,44]. Furthermore, studies have shown that the degree of cooperation could be inversely correlated with plaque index values [3,4,41].

We did not observe significant differences in the values of BOP between the RC and IC groups with regard to the type of treatment performed, i.e., surgical or non-surgical. Overall, the studies showed a substantial reduction in the percentage of sites with BOP following both treatment modalities, reflecting the systematic approach to plaque control incorporated in the studies [38,39].

The final logistic regression model for the recurrence of periodontitis in this study retained as significant variables smoking, diabetes, > 30% of BOP sites, PD 4–6 mm, irregular compliance, and surgical treatment.

Concurrent with our findings, previous studies in PMT have shown that smoking [13,27,34,45–47]and diabetes [4,13,48–50]are strong risk factors for recurrence of periodontitis and TL. Thus, the identification of factors, such as smoking, diabetes, and more severe disease, underscore the need for individual risk assessment of individuals in setting the frequency of visits for PMT [12,13,26].

A recent publication [51] indicated that non-compliance with periodontal maintenance cannot be solely explained by one determinant but rather might involve an individual’s health beliefs, emotional intelligence, psychological stressors, and personality traits [4]. Thus, it provides, for the health professional, a comprehensive view of the periodontally susceptible individual.

## Conclusion

In conclusion, irregular compliance and surgical procedures in subjects undergoing PMT presented higher rates of recurrence of periodontitis and TL when compared to regular compliant patients undergoing non-surgical procedures. This finding demonstrates the influence of compliance and treatment strategy during PMT on maintaining periodontal condition homeostasis.

## Supporting Information

S1 Dataset (ZIP)

S1 Table Characterization of the sample regarding variables of interest (n = 212) at final examination. (PDF)
