# Supplementary material for: Surgical and Non-Surgical Procedures Associated with Recurrence of Periodontitis in Periodontal Maintenance Therapy: 5-Year Prospective Study
Source: PLoS One. 2015 Oct 23;10(10):e0140847. doi: 10.1371/journal.pone.0140847 (PMC4619675; doi:10.1371/journal.pone.0140847)
Supplement: S1 Table — (PDF) [file pone.0140847.s002.pdf]

Table. Characterization of the sample regarding variables of interest (n=212) at final examination.

Characteristic	Regular Compliers		Irregular Compliers		p
	n=96	%	n=116	%	
**Gender***					
Female	56	58.7	52	44.8	
Male	40	41.3	64	55.2	**0.023**a
**Age groups (range 23 – 70 years)**					
Up to 40 years of age	15	15.4	21	18.3	0.660a
41 to 55 years of age	32	33.7	43	37.2	**0.046**a
> 55 years of age	49	50.9	52	44.5	0.930a
**Co-habitation status**					
With companion	75	78.3	83	71.2	0.084a
Without companion	21	21.7	33	28.8	
**Smoking**					
Non-smoker	71	73.9	83	71.6	0.972a
Smoker/former smoker	25	26.1	33	28.4	
**Diabetes**					
Yes	13	13.6	18	15.5	
No	83	86.4	98	84.5	0.088a
**Number of PMT Visits** (mean ± s.d)**	11.7 (±2.8)		5.4 (±1.3)		**0.023**b
**Time since APT/months (mean ± s.d)**	61.2 (±3.4)		62.6 (±3.9)		0.782b

PMT = Periodontal maintenance therapy; APT = Active periodontal therapy; [a]Chi-squared test, p = 0.023; [b]Student t test for independent samples, p < 0.019. Significant values are shown in bold.
